# Are scientific abstracts written in poetic verse an effective representation of the underlying research?

**DOI:** 10.12688/f1000research.7783.3

**Published:** 2016-08-25

**Authors:** Sam Illingworth

**Affiliations:** 1 School of Research, Enterprise & Innovation, Manchester Metropolitan University, Manchester, UK

**Keywords:** Science Communication, Interdisciplinary, Communication, Publishing

## Abstract

The central purpose of science is to explain (Purtill, 1970). However, who is that explanation for, and how is this explanation communicated once it has been deduced?

Scientific research is typically communicated via papers in journals, with an abstract presented as a summary of that explanation. However, in many instances they may be written in a manner which is non-communicatory to a lay reader (Halliday & Martin, 2003). This study begins to investigate if poetry could be used as an alternative form of communication, by first assessing if poetic verse is an effective form of communication to other scientists. In order to assess this suitability, a survey was conducted in which two different groups of participants were asked questions based on a scientific abstract. One group of participants was given the original scientific abstract, whilst the second group was instead given a poem written about the scientific study. Quantitative analysis found that whilst a scientific audience found a poetic interpretation of a scientific abstract to be no less interesting or inspiring than the original prose, they did find it to be less accessible. However, further qualitative analysis suggested that the poem did a good job in conveying a similar meaning to that presented in the original abstract. The results of this study indicate that whilst for a scientific audience poetry should not replace the prose abstract, it could be used alongside the original format to inspire the reader to find out more about the topic. Further research is needed to investigate the effectiveness of this approach for a non-expert audience.

Alternative version:

Are scientific papers understood,

By anyone from outside of the field;

And is an abstract really any good,

If jargon means its secrets aren’t revealed?

Could poetry present a different way,

Of summing up research in a nutshell;

Presented in a language for the lay,

Yet still useful for scientists as well?

This study aimed to find if it was true,

That poems could be a way to convey fact;

By splitting sample researchers in two:

And giving each a different abstract.

The findings showed that whilst prose was preferred,

Related meanings from both were inferred.

## Introduction

The central purpose of science is to explain ( Purtill, 1970). However, who is that explanation for, and how is this explanation communicated once it has been deduced? Scientific research is typically communicated via papers in journals, but whilst to an insider (i.e. a scientist in that field) these papers and journals represent an efficient and effective way of communicating research, to an outsider (i.e. a member of the general public) what they represent and report on may not be at all clear ( Meadows, 1985), and in many instances they may be written with a lexical density that makes them inaccessible to a lay reader ( Halliday & Martin, 2003).

Almost all journals require the authors to provide a word-limited abstract as part of the submission process, and whilst the specifics of these abstracts will vary from journal to journal, their purpose effectively remains the same, with Johnson (1995, pp. 28) defining them as “a concise representation of a document’s contents to enable the reader to determine its relevance to specific information.” If the central purpose of science were indeed to explain, is the central purpose of an abstract therefore a summary of that explanation? Swales (1990) considers a scientific abstract to be a ‘rite of passage’ for gaining entry into the scientific community, and that in order to do so the writer needs to demonstrate an “increasing mastery of the academic dialect” ( Orasan, 2001, pp. 2).

Andrade (2011, pp. 172) notes that “for the vast majority of readers, the paper does not exist beyond its abstract,” with the majority of researchers using the abstract to determine if the scientific study is relevant to them and worthy of a further investment of their time in reading it in its entirety. As noted by Fletcher (1988), the creation of an abstract is often also an extremely important process for clarifying the narrative of the scientific study in the mind of the author(s) themselves. Hartley (2003) also found that structured abstracts (i.e. those split into subheadings of: Background, Aims, Methods, Results, and Conclusions, or their equivalents) were found to be more informative and also provide greater clarity than their unstructured counterparts.

Whilst the exact format and structure of the abstract will be determined by the journal in which the article is to be published, the purpose of writing an abstract should be to extract and summarize ( Alexandrov & Hennerici, 2006), with the primary objective to not only provide information, but also to convince the reader to finish the remainder of the paper, which in some instances will involve paying for the privilege ( Koopman, 1997).

Orasan (2001) also observed that many authors of scientific papers do not consider the abstract to be particularly important, arguing that in many cases it is written as a necessity just before the paper is submitted. Is it therefore the case, that rather than being an effective and economical method of communicating the research, that the abstract instead represents a rushed précis of the research findings, with an even higher lexical density than that of the main body of the text? If so, then are they useful to anyone who might consider themselves, or indeed be considered an outsider? And if non-experts are unable to fully grasp the summary of the explanation, then what hope do they have of being able to fully understand the research and its potential relevance to them? Cross & Oppenheim (2006) also notes that there is probably little formal training in abstract writing, which is why in some instances there may be a lack of clarity in the abstracts that are produced in scientific journal articles.

Climate change research is a subject which has potential relevance on a global scale, however Rudiak-Gould (2014) found that whilst members of the general public receive information about climate change through the first-hand experience of its effects on their environment, it is still absolutely necessary to effectively communicate the science to them as well. This is because as well as the difficulty in objectively observing long-term trends, there are other issues in their day-to-day lives that the general public must concern themselves with as well. In their study Rudiak-Gould (2014) worked with the Marshall Island’s indigenous population, the Majuro people, where more pressing concerns than long-term sea-level rise were short-term anxieties related to e.g. fluctuations in the cost of rice. This argument is relevant in other communities, where issues such as job security, energy prices and mortgage rates might well take precedence over concerns relating to climate change. In other words, it is not simply enough to assume that people will take notice of their changing environments and act upon them; instead there needs to be an intervention in terms of the effective communication of what is happening, the consequences of this, and what can be done in order to mitigate the effects. Effective science communication should “facilitate conversations with the public that recognize, respect, and incorporate differences in knowledge, values, perspectives, and goals.” ( Nisbet & Scheufele, 2009, pp. 1767).

The accurate communication of scientific research is also vital so that the general public are aware of the consensus in terms of scientific understanding, and researchers should not forget the ‘moral dimension’ and sense of responsibility in terms of communicating their findings to others ( Tickell, 2002). This is particularly prominent for studies discussing the anthropogenic nature of climate change, with John *et al.* (2013) finding that over 97% of climate change papers published between 1991 and 2011 agreed that climate change is a human-caused phenomenon. However, this is not always the way that this argument is presented, which is why it is absolutely vital that scientists endeavour to make their research as transparent and accessible as possible. It has also been shown that the polarization over the validity of climate change science is reduced when information content is communicated alongside a consideration for cultural meanings ( Kahan *et al.*, 2015). Can scientific articles take into account these cultural values, thereby acting as an effective way of communicating information? Likewise, if journals are to act as an effectual conduit between scientists and the general public, then how can abstracts be constructed so as to appeal to the widest possible audience whilst still conveying useful and meaningful information, and is there a medium that can be exploited in order to ease this transition?

The former United States Poet Laureate Robert Pinsky writes that ( Pinsky, 2009, pp. 46):

“Poetry mediates, on a particular and immensely valuable level between the inner consciousness of the individual reader and the outer world of other people.”

Similarly, the English romantic-era poet Percy Bysshe Shelley noted that “poetry lifts the veil from the hidden beauty of the world” ( Shelley, 1888). Could poetry therefore be the medium with which to help encapsulate non-expert audiences with research findings? Science and poetry have much in common, both in terms of their use of metaphor and their embodiment of process (see e.g. Illingworth, 2016), and as the American poet Robert Kelly noted in his poem ‘Science’ ( Kelly, 2007):

Science explains nothing
  but holds all together
  as many things as it can count
science is a basket
  not a religion he said
  a cat as big as a cat
the moon the size of the moon
  science is the same as poetry
  only it uses the wrong words.

Could poetry therefore help scientists to choose their words more carefully, thereby helping them to avoid the academic dialect that so often ostracizes the non-expert? There is in fact a historical precedent for science being written in poetic verse, most famously evidenced by the works of Erasmus Darwin (1798). However, rather than an entire journal article penned in poetic verse, might instead their abstracts be written in this style, and in doing so might they then appeal to a wider audience, be more readily understood, and potentially encourage the reader to investigate the topic further?

If poetry is to be used to help better communicate scientific abstracts to the general public however, it is first of all important to establish if this form of communique is still useful to the experts in the field. In other words, if poetic verse were to be accepted as a suitable abstract style, then would this still be accessible and informative to other scientists? It is the purpose of this study to determine if this new format means that the abstract is still a useful commodity to the ‘insiders.’

This paper is organised as follows: the methodology used in this study is described, followed by a presentation and discussion of the results from this study. Finally, some perspectives on this work are outlined, discussing what the results imply for future work and for the scientific abstract in general.

## Survey selection

In order to assess the suitability of using poetry in scientific abstracts, a survey was conducted in which two different groups of participants were given an abstract relating to a scientific paper, before being asked questions based on this abstract. One of the groups of participants was given the original scientific abstract, whilst the second group were given a poem that was written about the scientific study. Apart from this the two surveys were identical, and the survey was conducted using the free online survey builder ‘Typeform’ ( www.typeform.com), comprising of nine questions delivered with a mixed-method approach. Of these nine questions, five of them asked for demographic information, whilst the remaining four were all related to the abstract itself, asking the participants to sum up in their own words what they thought that the research was about, and also asking them to mark the abstract out of ten (zero being the least) for how accessible and interesting they found the abstract, as well as how likely they were to go and find out more about this research as a result of reading the abstract. A copy of the questionnaire can be found in the Supplementary Materials section of this article, and the non-demographic questions are given below (please note that the numbering of these questions is different to how they appeared in the survey):

Q1 How accessible did you find the abstract? (mark from 0 to 10, with 0 being the least)
Q2 How interesting did you find the abstract? (mark from 0 to 10, with 0 being the least)
Q3 As a result of reading the abstract, how likely are you to go and find out more about this research? (mark from 0 to 10, with 0 being the least)
Q4 After reading the abstract, what do you think that this research is about? (open-ended)

The choice of the abstract and poem themselves was important, as it was necessary to choose an abstract that was well written so as not to bias the results of the study, it was also important to choose a topic that would be of potential relevance to non-experts. As discussed in the introduction, research concerning climate change demands to be communicated, because of its global relevance and the potential societal consequences of its findings. Ideally then, the scientific study in question would be related to climate change, and would have a well-written abstract.

It is also important to consider the issue of transformation, and how this potentially affects the goal of the abstract. As discussed above, the primary objective of the abstract is to both provide information and also to convince the reader to finish the remainder of the paper. The nature of the poem means that it is more likely to be thought of as a popular piece of science writing rather than a professional piece of science writing, as is the case for the original abstract. As such, care must be taken to ensure that in the transformation from prose into poetry, the primary objective does not also fully transform into that of purely establishing the novelty of the topic ( Rowan, 1989). In order for the poem to still be useful to scientists it must still provide a useful summary of the research. However, could the poem do so in a more accessible manner than that of the more traditional scientific abstract? It is also important to consider that scientific discourse and literature have traditionally differing goals, with science designed to provide empirical support, and poetry (as a form of literary discourse) designed mainly to entertain (see e.g. Rowan, 1988; Rowan, 2003). With this in mind, this study sets out in part to see if poetry can in fact provide information about the natural world, by accessibly summarising a piece of scientific research.

I write a weekly blog ( http://thepoetryofscience.scienceblog.com/), in which I communicate recent scientific research to the general public by reading journal articles and then writing a poem that summarises these findings. From the archive of these poems, there was one which was written about a study into the projected deglaciation of western Canada ( Clarke *et al.*, 2015). This study related to climate change, was extremely well written, and was published in a very reputable journal. Glaciers represent natural hazards to local communities and beyond because of their importance to regional water resources ( Marshall, 2014), as well as the danger that they pose in relation to outburst floods brought about by a warming climate (see e.g. Bolch *et al.*, 2008). The communication of the impact of climate change on glacial retreat is therefore important, not only for those downstream of the glaciers themselves ( Vuille *et al.*, 2008), but also for the wider global communities that are affected by the changes to the global carbon budget and ocean circulation that can be brought about my glacial change ( Piotrowski *et al.*, 2005). The abstract for the Clarke *et al.* (2015) paper, as well as the accompanying poem were thus chosen for this study. It was also important that the abstract that was chosen was itself well written, and that it met the primary objectives of an abstract that was described in the Introduction, i.e. that it presented a clear and accurate summary of the paper, and left the reader wanted to find out more. Whilst this latter point is relatively subjective, it was the author’s opinion that this abstract did indeed meet these primary objectives, thus the reason for its selection. If a less clear or less obviously enticing abstract had been chosen then there was a risk that the study might be being perceived as negatively biased towards the prose version of the abstract. It is acknowledged that in choosing such an effective abstract the study might instead by positively biased towards the original prose, but given the nature of the investigation it was felt that this was more appropriate.

Given that this study aimed to provide an initial insight into the plausibility of using poetic verse in scientific abstracts, a total sampling size of 100 participants (50 for each survey) was chosen. A convenience sampling strategy was adopted, in which the survey was advertised using Twitter, via both multiple tweets from the author’s Twitter account and the re-tweets that also resulted from this. The target audience were people that identified themselves as being scientists or who had a scientific background, which for the sake of this study were taken to be people that had achieved at least an undergraduate degree in science. This study was carried out according to the British Educational Research Association’s (BERA) ethical guidelines for educational research, with all of the data in this study fully anonymised.

The abstract from the Clarke *et al.* (2015) study that was given to the ‘prose’ group of the participants is shown below:

“Retreat of mountain glaciers is a significant contributor to sea-level rise and a potential threat to human populations through impacts on water availability and regional hydrology. Like most of Earth’s mountain glaciers, those in western North America are experiencing rapid mass loss. Projections of future large-scale mass change are based on surface mass balance models that are open to criticism, because they ignore or greatly simplify glacier physics. Here we use a high-resolution regional glaciation model, developed by coupling physics-based ice dynamics with a surface mass balance model, to project the fate of glaciers in western Canada. We use twenty-first-century climate scenarios from an ensemble of global climate models in our simulations; the results indicate that by 2100, the volume of glacier ice in western Canada will shrink by 70 ± 10% relative to 2005. According to our simulations, few glaciers will remain in the Interior and Rockies regions, but maritime glaciers, in particular those in northwestern British Columbia, will survive in a diminished state. We project the maximum rate of ice volume loss, corresponding to peak input of deglacial meltwater to streams and rivers, to occur around 2020–2040. Potential implications include impacts on aquatic ecosystems, agriculture, forestry, alpine tourism and water quality.”

Whilst the poem that was distributed to the ‘poetry’ group is as follows:

In Canada a study found,
  How glaciers melt in the West.
  The shrinkage is beyond profound,
  With seventy per cent at best;
  If we ignore the Earth’s request
  Then ninety-five per cent will go.
  New barren lands will not be dressed,
  With climate change too warm for snow,
  The alpine streams and sapphire lakes they too will go.

Once the responses to the surveys were collected, the quantitative outcomes were assessed, and the qualitative analysis tool NVivo (Version 10.2.2) was used to perform a qualitative thematic analysis. These results are presented and discussed in the following section.

## Results and discussion

Answers to poetry and prose surveys These are the responses to the survey that was used in this study to assess the effectiveness of poetry as a form of scientific abstract. Click here for additional data file. Copyright: © 2016 Illingworth S 2016 Data associated with the article are available under the terms of the Creative Commons Zero "No rights reserved" data waiver (CC0 1.0 Public domain dedication).

**Figure 1.**
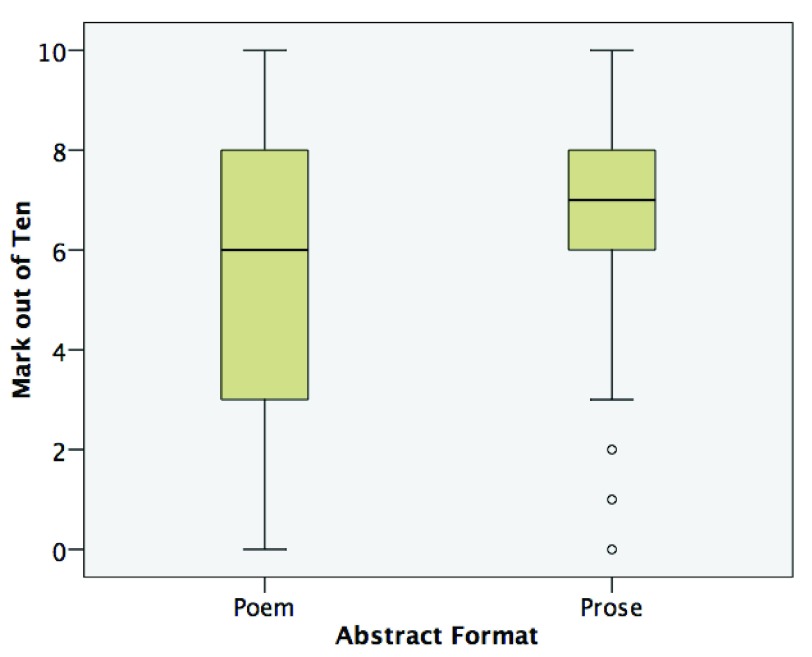
Box-plot of the responses to the survey Q1, which asked “How accessible did you find the abstract (0 is least)?” Outliers are represented by white circles.

Box plots of marks out of ten for the responses to the three quantitative survey questions (Q1–Q3) are given in Figure 1– Figure 3, whilst Figure 4 shows the number of words that were written by each of the participants in response to the open-ended qualitative survey question (Q4). As can be seen from Figure 1– Figure 3, the abstracts that were written in the traditional prose format seemed to receive higher marks than their poetic equivalents in terms of accessibility, interest and inspiration (i.e. the likelihood of the reader wanting to find out more about the research topic). Figure 4 also suggests that the average number of words that were written in response to what the scientific study was about was also much higher for the prose group. These differences can also be seen from the mean values that are presented in Table 1. However, in order to ascertain that there really is a marked difference in the average responses to the survey questions, it is necessary to carry out a statistical test to ensure that this is the case.

**Figure 2.**
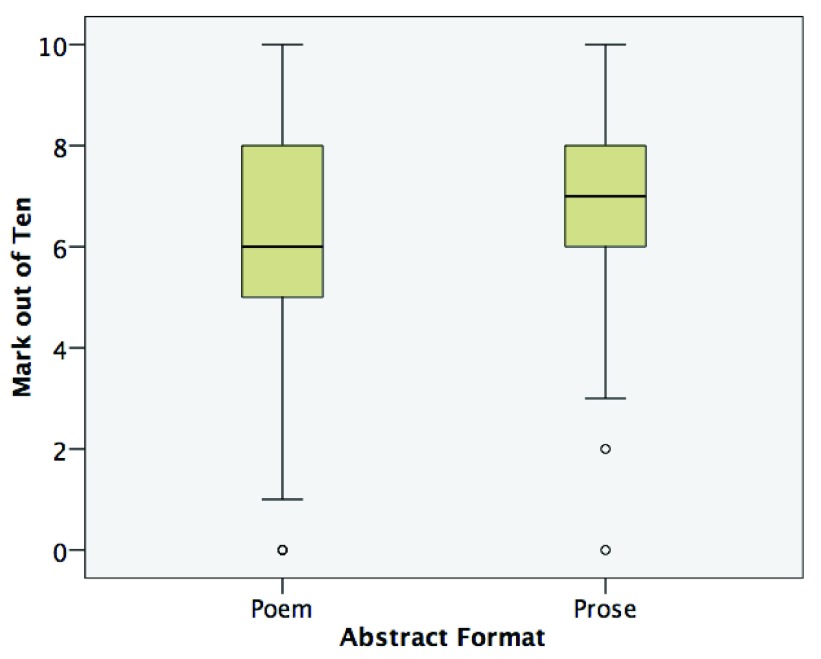
Box-plot of the responses to survey Q2, which asked "How interesting did you find the abstract (0 is least)?” Outliers are represented by white circles.

**Figure 3.**
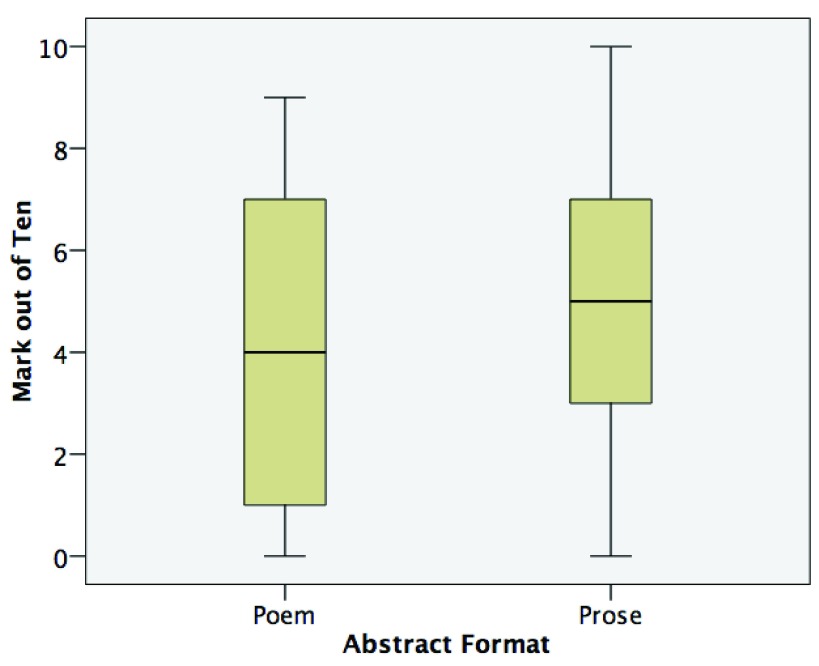
Box-plot of the responses to survey Q3, which asked "As a result of reading the abstract, how likely are you to go and find out more about this research (0 is least)?”

**Figure 4.**
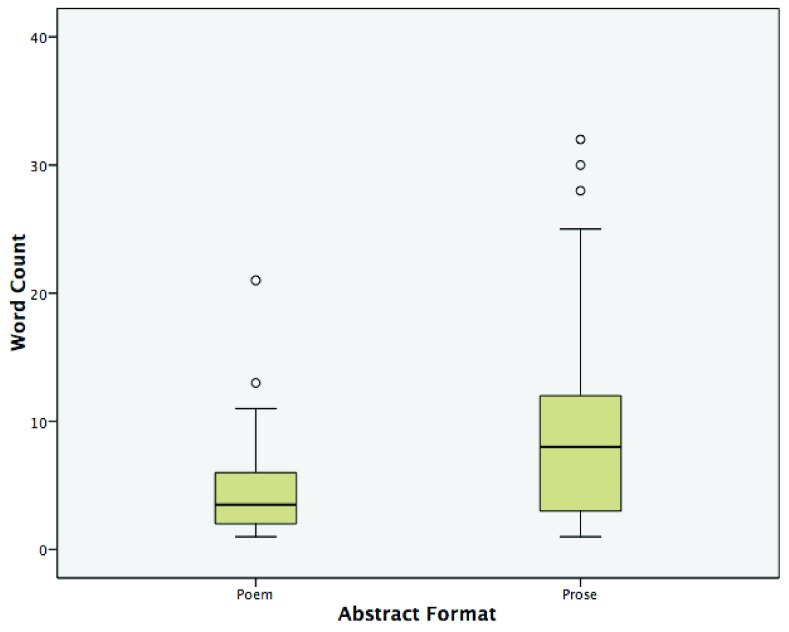
Box-plot of the number of words used in the survey Q4, which asked “After reading the abstract, what do you think that this research is about?”. Outliers are represented by white circles.

**Table 1.** Summary of quantitative data. The first column corresponds to the survey questions, and the second and third columns give the median for the poem and prose groups, respectively. The fourth column gives the asymptotic significance (2-tailed) p-value for the Mann-Whitney U test.

	Median value		Statistical test
Category	Poem	Prose	Asymp. sig. (2-tailed)
**Q1: Accessibility**	6	7	0.015
**Q2: Interest**	6	7	0.106
**Q3: Inspiration**	4	5	0.340
**Q4: Word count**	3.5	8	0.002

Questions 1–3 are based on an ordinal scale from 0 to 10 (where 0 was the lowest response), as such the responses to these questions will not be normally distributed, and it is therefore necessary to use nonparametric statistics, which make no assumptions about the probability distributions of the variables that are being assessed. Regarding the word count associated with Q4, and because the sample size was relatively small, the Shapiro-Wilk test was used to test for normalisation in the data. For the responses to both the poetry and the prose abstracts it was found that the p-value of the Shapiro-Wilk test was less than 0.001, therefore suggesting that the data significantly deviates from a normal distribution. As such, a nonparametric statistical test was also needed to check if the average word count in the response to Q4 was statistically different between the prose and the poetry groups.

Statistical analysis was performed using the Mann-Whitney U-test, where a p-value of less than 0.05 is considered statistically significant, i.e. there is a statistically significant difference between the responses of the two groups. The Mann-Whitney U-test is the nonparametric equivalent of the independent t-test, and was conducted using IBM SPSS (Version 22.0), the results of which are shown in Table 1. As can be seen from Table 1, at the 95% confidence level the prose group found the abstract more accessible than the poetry group. Similarly, the prose group were statistically more likely to write more than the poetry group in their responses to Q4. With regards to the interest and inspiration of the abstracts, there was no statistically significant difference between the two groups at the 95% confidence level, with the p-values being 0.106 and 0.34, respectively.

From the results of the surveys, the prose abstract would appear to be more accessible than the poetry version, with both generating similar levels of interest and inspiration. Given that this research aims to see if abstracts in poetic form are still useful to scientists, from the results of the survey it would appear that they are less useful than a well written piece of prose. It is also worth noting that that the median mark for Q3 (i.e. the extent to which having read the abstract, the reader was inclined to go and find out more about the subject) was below 5 for both formats. It is also a little surprising that the readers found the poem to be no more interesting or inspirational than the abstract, but again this might be down to the high quality of the prose, or alternatively a reflection on the quality of the poem! This would also suggest that a poetic version of the abstract would not serve to further entice the reader to pursue (or in some instances purchase) the remainder of the article.

So, from a statistical analysis of the survey it would appear that poetry should not be used as an alternative to prose in the presentation of scientific abstracts, as scientists find this approach to be less accessible. However, before making any further analysis, it is first necessary to carry out a qualitative assessment of the responses to Q4. Could it be that despite ranking the poetic form as being less accessible than the standard format, the participants were still able to successfully deduce the main focus of the research?

From the responses to Q4, the qualitative analysis tool NVivo was used to perform a qualitative thematic analysis. An open coding approach was adopted, in which a number of major categories were selected based on the participant’s responses. The responses were then re-examined in order to confirm that the major categories were an accurate representation of the responses. This methodology was adopted for responses from both the poetry and the prose groups, and was carried out until there were no further themes found to be emerging, i.e. until descriptive saturation was reached. Only categories which received a total of more than five responses were considered for analysis. The different major themes, along with the corresponding coding frequencies, are shown in Table 2, whilst Table 3 gives a more detailed description of each of the categories.

**Table 2.** Major categories for responses to Q4, colour coded according to frequency, on a red-green colour scale with red being the least frequent and green being the most.

	Category								
Group	Glaciers	Climate change	Global warming	Location	Impacts	Future	Model	Ice	Environment
Poetry abstract	18	19	15	10	8	2	0	5	3
Prose Abstract	39	13	5	9	10	14	13	4	5

**Table 3.** Descriptions of the different categories used in the qualitative coding of the responses to Q4.

Category	Description
**Glaciers**	The response explicitly mentions glaciers.
**Climate change**	The response explicitly mentions Climate Change.
**Global warming**	The response explicitly mentions Global Warming.
**Location**	The response explicitly mentions a specific location (e.g. Canada or North America).
**Impacts**	The response explicitly mentions the word ‘impact’ or ‘effect’.
**Future**	The response explicitly makes reference to what things might look like in the future.
**Model**	The response explicitly mentions models/modelling.
**Ice**	The response explicitly mentions ice, as opposed to, or in addition to, glaciers.
**Environment**	The response explicitly mentions the environment.

The median number of categories contained within each response for the poetry and prose groups were 1 and 2, respectively. The Mann-Whitney U-test was used to compare these values, with an asymptotic 2-tailed p-value of 0.011 indicating that at the 95% confidence interval the responses from the prose group had statistically more category groupings per response than those from the poetry group. This result correlates well with the increased word count in Q4 for the prose group, thereby indicating that this group provided more verbose and detailed summaries than the poetry group.

In terms of the categories themselves, the only category that was applicable to only one group was the ‘Model’ category. This is not surprising, as the poem itself makes no mention of the fact that the study in question was based largely around a set of modelling simulations. This is perhaps a failing of the poem, but what is also interesting is that only 13 of the 50 participants (26%) in the prose group mentioned modelling, even though this is stated several times in the original abstract. Similarly, as can be seen from Table 2, only two of the respondents from the poetry group reference the ‘Future’, whereas 14 of the participants from the prose group make reference to this fact. Whilst it could be argued that ‘Climate change’ might imply a future event, the respondents in the ‘Future’ category made explicit reference to a future scenario. Given that the poem talks about what may happen in the future, it was surprising to see that only 4% of the respondents picked up on this. Similarly, the fact that only 28% of participants from the prose group made reference to this was lower than might have been expected.

Regarding the ‘Location’, ‘Impacts’, ‘Ice’ and ‘Environment’ categories, the proportion of respondents was almost identical. Of these, the relatively low number of responses in the ‘Location’ category from both the poetry and the prose groups (20% and 18%, respectively) was perhaps the most surprising, as both abstracts are very explicit in their description of Canada being the location of this study. It is perhaps not surprising that there are more references to ‘Climate change’ in the poetry group than in the prose group (38% compared to 26%), as the poem uses this phrase explicitly, whereas it is only implied in the original abstract. What is more unexpected is that the poetry group make more reference to ‘Global warming’ than the prose group (30% compared to 10%), even though the phrase itself appears in neither version of the abstract. Perhaps it is certain words in the poem like ‘barren’ and ‘melt’ that ellicit this response. It is also surprising that there is such a large difference between the two groups in terms of the ‘Glaciers’ category, with 78% of the prose group including mention of them in their response to Q4, compared to only 36% in the poetry group, given that this term is used in both versions. However, this is probably explained by the fact that the prose version of the abstract mentions the word ‘glacier’ or ‘glaciers’ seven times, in comparison to the solitary use of the word ‘glaciers’ in the poem.

In addition to the categories that are shown in Table 2, there are also some individual responses that are worth noting. Out of all of the responses, only one respondent, from the poetry group, had no comment. Similarly, only one respondent, this time from the prose group, commented that it was unclear from the abstract what the research was about. This would seem to indicate that despite the participants not necessarily being experts in this field, their scientific background meant that they were suitable subjects for the study. Two respondents from the poetry group responded at a meta level, with one simply writing ‘poetry’ in their response to Q4, and the other making reference to the grammar of the poem. In regards to emotive responses, only one of the respondents from the poetry group made reference to this, noting that the research was about “Melting Canadian glaciers, with projections for the future (if slightly emotive!)”.

As well as not conveying some key elements of the study (as was the case with the ‘Model’ category) there is also the danger that the poem might ellicit in the reader an implied meaning, which is present in neither the prose abstract nor the underlying research itself. However, it is encouraging that there were no major categories that were exclusive to the poetry group alone. The results of this qualitative analysis would therefore seem to suggest that on this occasion the poem did a good job in conveying a similar meaning to that presented in the original abstract, other than the omission of the modelling aspect of the research. It is worth noting that this category was also absent from the vast majority (74%) of the responses from the prose group.

The quantitative and qualitative analysis would seem to suggest that scientists find a well-written abstract written in prose format to be more accessible than its poetic equivalent. Similarly, they are more likely to write longer, and more detailed summaries about what they understand the research to be about. However, these summaries were found to be fairly similar between the two groups.

One final comparison that is worth noting is the length of the abstracts themselves. The poem is 58 words long, whereas the original abstract consists of 204 words. Could it be that the longer length of the prose abstract, combined with the expectancy of the readers in terms of what a scientific abstract should look like, meant that the prose format was able to convey more information and was therefore more accessible, encouraging more verbose and detailed summaries from the participants? Could it also be that the participants were on the whole less experienced in analysing poetry, and therefore felt less confident in elucidating on their opinions as to the nature of the poem? Whilst the poem is shorter than the prose and contains less statistical information, does that necessarily mean that it contains less detail? Could it be that instead of explicitly communicating detail (as is the case with the prose abstract), the poem instead had the affect of implying detail via an emotive response or reflection by the reader? That only one of the participants commented on the emotive nature of the poem suggests that in this instance it may not be the case, and that for the participants of this study there probably was a perceived lack of detail in the poem compared to the prose. However, as this was not commented on (nor asked by the survey) explicitly this cannot be confirmed.

As discussed in the Introduction, the very nature of this study involves transforming the abstract in some way, and whilst every effort has been made in this transformation to retain the information of the original abstract, it is clear from an analysis of the surveyed responses that this has not been entirely successful. This is most clearly evidenced with the omission of the word ‘model’ from the poem. Whilst it is likely that the results would have been different had the first line of the poem been replaced with “In Canada a model found,” this is not the transformation that the author decided upon. Has the text’s primary goal therefore also changed? As discussed in the Introduction, the primary goal of the scientific abstract is to offer an effective summary of the study, but also to compel the reader, in this instance to read the rest of the article. The results of the survey would suggest that rather than a transformation of primary goal, there has instead been a transformation of focus. As with the original abstract, the purpose of the poem was still to inform *and* entice scientists, by presenting an indicative summary of the underlying research, and also serving as a compelling case that the remainder of the article was worthy of the reader’s attention. The analysis of the qualitative data would seem to suggest that the poem has still served that purpose (seemingly neither improving nor diminishing the desire to find out more about the study), however the focus of the summary has undoubtedly shifted.

## Conclusion

This study presented itself as an investigation into whether poetry could be used as an alternative form of abstract in scientific journals. The rationale was that poetry might be a more effective and easily accessible format in terms of communicating the scientific findings to a non-expert audience, but that in order for this methodology to be considered it was first necessary to try and determine if replacing a traditional abstract with a poem would still be useful to scientists who were reading the article.

The quantitative analysis of the survey reveals that whilst a scientific audience find a poetic interpretation of a scientific abstract to be no more interesting or inspiring than the original prose, they do find it less accessible. This suggests that scientists are happier in reading a prose abstract, probably because they have more experience in doing so, but maybe also because the notion of reading a poetic abstract might conjure up images of having to write such an interpretation themselves! From the qualitative analysis, the interpretation by the two groups was similar, with the notable exception of the importance of modelling in the scientific study, which was absent from the poem. This would seem to suggest that for future studies a more suitable approach would be for the poem to be first peer-reviewed by the author of the original scientific study, in order to make sure that there were no omissions in terms of content, or indeed any additional inferences that were present due to an overly liberal artistic license.

The issue of transformation that was raised in the discussions is notable, and is certainly worthy of further investigation in future studies. However, such studies would need to be designed so as to specifically address this issue. For example, participants could be asked for their interpretations of the primary objective of each version of the abstract (poetry or prose). Alternatively, participants could be shown both versions of the abstract and could be asked to comment on similarities and differences between the two, both literally and in terms of what they convey.

Further research is needed to improve the practice of communicating science (see e.g. Treise & Weigold, 2002), and by investigating alternative methods of communications it is also possible to determine which areas are effective, and which require the most improvement. For example, from the results of this study, the scientific audience found the original abstract to be reasonably accessible and interesting, but they were not particularly inspired to go and find out more about the research. Could poetry therefore be written with a focus on inducing inspiration in the reader, to be read alongside the original abstract, which would still provide the accessibility required?

From the results of this research, a future study is now planned to investigate the effectiveness of abstracts in poetic form for a non-expert audience. Following some very useful suggestions from one of the reviewers of this manuscript, such a study would benefit from using three versions of the scientific abstract: the original prose abstract (designed for expert audiences); a second abstract written in prose for a non-expert audience; and a third abstract, written as a poem. As noted above, it would also be important in such a study to include a variety of different poems. In order to give the most robust dataset, it is also planned for a number of different scientific abstracts (and the accompanying non-expert prose abstracts and poetic abstracts) to be used in this future study. Depending on the results of that study it might be that poetic abstracts could be offered as an alternative abstract, to sit alongside the more traditional prose format. However, from a scientific point of view the results presented here suggest that poetry alone is not an effective representation of the underlying research in a scientific study.

It is acknowledged that the results and subsequent analysis that are presented here represent the responses from only one study that was carried out on a small subset of participants. As such, it is important to recognise the limitations of the findings, and to allow that a different set of results may be evident if a different group of participants were surveyed. Likewise, the responses of the participants would probably be different if they were shown different poetical interpretations (from the same or different authors) of the same abstract. Given these limitations, I hope that this study has demonstrated that there is a capacity and a merit in such investigations, and also that it has served as inspiration for future work.

## Data availability

The data referenced by this article are under copyright with the following copyright statement: Copyright: © 2016 Illingworth S

Data associated with the article are available under the terms of the Creative Commons Zero "No rights reserved" data waiver (CC0 1.0 Public domain dedication). 

F1000Research: Dataset 1. Answers to poetry and prose surveys, 10.5256/f1000research.7783.d111682 ( Illingworth, 2016).

## Supplementary Material

**Survey 1: poetry group.**

**Survey 2: prose group.**
