# Supplementary material for: Are scientific abstracts written in poetic verse an effective representation of the underlying research?
Source: F1000Res. 2016 Aug 25;5:91. Originally published 2016 Jan 21. [Version 3] doi: 10.12688/f1000research.7783.3 (PMC5017291; doi:10.12688/f1000research.7783.3)
Supplement: Supplementary file 2 [file f1000research-5-10229-s0001.tgz › fc27184d-8329-47fa-b376-fc75d187e92f.pdf]

1 What is your gender? *

2 What is your age? *

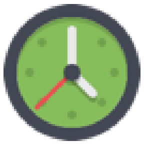

◯ 17 or younger ◯ 18 to 24 ◯ 25 to 34 ◯ 35 to 44 ◯ 45 to 54 ◯ 55 to 64
◯ 65 to 74 ◯ 75 or older ◯ I prefer not to answer

3 In which country were you born? *

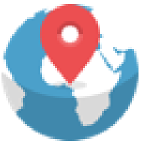

Ooops! You must make a selection

4 What is the highest level of science education you have completed? *

◯ Less than High School ◯ High School or equivalent ◯ Attended College / University
◯ Associate degree ◯ Bachelor's degree ◯ Master's degree ◯ Professional degree
◯ Doctorate degree ◯ I prefer not to answer

5 Which industry do you work in or are most involved with? *

Ooops! You must make a selection

In Canada a study found,
How glaciers melt in the West.
The shrinkage is beyond profound,
With seventy per cent at best;
If we ignore the Earth's request
Then ninety five per cent will go.
New barren lands will not be dressed,
With climate change too warm for snow,
The alpine streams and sapphire lakes they too will go.

Please read this abstract and then answer the proceeding questions.

6 After reading the abstract, what do you think that this research is about? *

How accessible did you find the abstract? (0 is least) *

0 1 2 3 4 5 6 7 8 9 10

How interesting did you find the abstract? (0 is least) *

0 1 2 3 4 5 6 7 8 9 10

As a result of reading the abstract, how likely are you to go and find out more about this research? (0 is least) *

0 1 2 3 4 5 6 7 8 9 10

Submit

Never submit passwords! - Report abuse
