# Supplementary material for: Are scientific abstracts written in poetic verse an effective representation of the underlying research?
Source: F1000Res. 2016 Aug 25;5:91. Originally published 2016 Jan 21. [Version 3] doi: 10.12688/f1000research.7783.3 (PMC5017291; doi:10.12688/f1000research.7783.3)
Supplement: Supplementary file 3 [file f1000research-5-10229-s0002.tgz › 37f22509-5cfc-41f1-9a40-b2016c484ea5.pdf]

1 What is your gender? *

2 What is your age? *

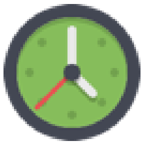

◯ 17 or younger ◯ 18 to 24 ◯ 25 to 34 ◯ 35 to 44 ◯ 45 to 54 ◯ 55 to 64
◯ 65 to 74 ◯ 75 or older ◯ I prefer not to answer

3 In which country were you born? *

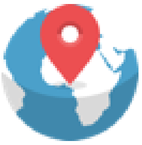

Ooops! You must make a selection

4 What is the highest level of science education you have completed? *

◯ Less than High School ◯ High School or equivalent ◯ Attended College / University
◯ Associate degree ◯ Bachelor's degree ◯ Master's degree ◯ Professional degree
◯ Doctorate degree ◯ I prefer not to answer

5 Which industry do you work in or are most involved with? *

Ooops! You must make a selection

Retreat of mountain glaciers is a significant contributor to sea-level rise and a potential threat to human populations through impacts on water availability and regional hydrology. Like most of Earth's mountain glaciers, those in western North America are experiencing rapid mass loss. Projections of future large-scale mass change are based on surface mass balance models that are open to criticism, because they ignore or greatly simplify glacier physics. Here we use a high-resolution regional glaciation model, developed by coupling physics-based ice dynamics with a surface mass balance model, to project the fate of glaciers in western Canada. We use twenty-first-century climate scenarios from an ensemble of global climate models in our simulations; the results indicate that by 2100, the volume of glacier ice in western Canada will shrink by 70 ± 10% relative to 2005. According to our simulations, few glaciers will remain in the Interior and Rockies regions, but maritime glaciers, in particular those in northwestern British Columbia, will survive in a diminished state. We project the maximum rate of ice volume loss, corresponding to peak input of deglacial meltwater to streams and rivers, to occur around 2020–2040. Potential implications include impacts on aquatic ecosystems, agriculture, forestry, alpine tourism and water quality.

Please read this abstract and then answer the proceeding questions.

6 After reading the abstract, what do you think that this research is about? (0 is least) *

How accessible did you find the abstract? (0 is least) *

○ 0 ○ 1 ○ 2 ○ 3 ○ 4 ○ 5 ○ 6 ○ 7 ○ 8 ○ 9 ○ 10

How interesting did you find the abstract? (0 is least) *

○ 0 ○ 1 ○ 2 ○ 3 ○ 4 ○ 5 ○ 6 ○ 7 ○ 8 ○ 9 ○ 10

As a result of reading the abstract, how likely are you to go and find out more about this research? *

○	○	○	○	○	○	○	○	○	○	○
0	1	2	3	4	5	6	7	8	9	10

Submit

Never submit passwords! - Report abuse
