# Supplementary material for: Are scientific abstracts written in poetic verse an effective representation of the underlying research?
Source: F1000Res. 2016 Aug 25;5:91. Originally published 2016 Jan 21. [Version 3] doi: 10.12688/f1000research.7783.3 (PMC5017291; doi:10.12688/f1000research.7783.3)
Supplement: Peer review history

# Peer review: Referee response for version 3

**DOI:** 10.5256/f1000research.10229.r15881

**Published:** 2016-10-03

**Authors:** Magnus Johnson

**Affiliations:** 1 Centre for Environmental and Marine Sciences (CEMS), University of Hull, Scarborough Campus, Scarborough, UK

This article looks fine now.  The author has responded well to the comments of reviewers.

I have read this submission. I believe that I have an appropriate level of expertise to confirm that it is of an acceptable scientific standard.

# Peer review: Referee response for version 3

**DOI:** 10.5256/f1000research.10229.r15883

**Published:** 2016-09-19

**Authors:** Laura Bowater

**Affiliations:** 1 Norwich Medical School, University of East Anglia, Norwich, UK

I am happy to approve this article for indexing. All my reservations have been addressed by the author.

I have read this submission. I believe that I have an appropriate level of expertise to confirm that it is of an acceptable scientific standard.

# Peer review: Referee response for version 2

**DOI:** 10.5256/f1000research.9197.r15242

**Published:** 2016-08-08

**Authors:** Laura Bowater

**Affiliations:** 1 Norwich Medical School, University of East Anglia, Norwich, UK

I found this an interesting and an unusual article, and although I feel I might have predicted the key finding from my own perspective as a representative of the scientific community it is a worthwhile research project to find the evidence to back up my own conjectures.

 I was surprised to find that this article did not contain an abstract that was delivered in poetry as oppose to prose or at least had two versions of the abstract. 

 Within the introduction, I enjoyed reading about the power and placement of a research article and what it represents to the reader. As the author suggests the abstract is often the decision-making part of the article: do you read further or purchase the article or do you pass it by, and, I know in myself that I have often been guilty of regarding the abstract as the last step prior to submission. To me, the power of this article has been to remind me to spend more time on this aspect of writing a research paper. 

 Within the discussion and the conclusions, the concept, that poetry may serve as a useful abstract I still have some outstanding questions that I would like to have addressed. First of all, did the author consider producing two or three versions of the poem? The poem did seem to be less factually intense than the original abstract and I wonder if the authors feel that this may be reflected in the views of the scientists. I am also aware that the beauty of a poem often lies in the critical analysis that underpins the choice of word and form used by the author. This is a skill that can be developed often throughout a school of HE education and I am not sure that many scientists will have received that training. However again this is my conjecture and not necessarily based on fact. The suggestion is that scientists are happier to read a prose abstract. I also wonder if this also reflects many scientists concern with an issue of whether they might have to learn to write an abstract as a poem. 

 I agree with the author that the abstract chosen was well written and accessible. I think that this is the key. Prose or poetry is not the issue but accessibility is and writing that can be clearly understood has to be key to this. The author suggests that they would like to repeat the  study of a more 'general' audience. I can see the value of this approach and the importance of this study as it is important to explore how science can be made more accessible to a more general public. I wonder whether the author would consider using three versions of an abstract in future studies. The first an abstract written for a scientific community but not necessarily a more general audience, the second an abstract that is written in a clear accessible language and the last a poem. I also think that a lot more value would be placed in having a variety of abstracts presented in this way to get a more robust data set. I was also looking for a more thorough explanation for why the author only chose to present one poetry and prose abstract within this study.

 For my own use, how I would use this article in the future is to consider bringing this into my own teaching practice. I think that a student that can read and summarise an article effectively in a poem may be demonstrating some key academic skills that include reading and digesting an academic piece of writing, being able to summarise effectively, being able to use effective writing to write for a less scientifically literate audience.

I have read this submission. I believe that I have an appropriate level of expertise to confirm that it is of an acceptable scientific standard, however I have significant reservations, as outlined above.

# Response

**Published:** 2016-08-18

**Authors:** Samuel Illingworth

**Affiliations:** Manchester Metropolitan University, UK

*I found this an interesting and an unusual article, and although I feel I might have predicted the key finding from my own perspective as a representative of the scientific community it is a worthwhile research project to find the evidence to back up my own conjectures.*

 Thank you for these kind comments, and also for the rest of the review that you have provided, and which I now respond to on a point-by-point basis.

*I was surprised to find that this article did not contain an abstract that was delivered in poetry as oppose to prose or at least had two versions of the abstract.*

 This is an excellent point. An alternative abstract in the form of a poem has now been provided. J

 Are scientific papers understood,

 By anyone from outside of the field;

 And is an abstract really any good,

 If jargon means its secrets aren’t revealed?

 Could poetry present a different way,

 Of summing up research in a nutshell;  

 Presented in a language for the lay,

 Yet still useful for scientists as well?

 This study aimed to find if it was true,

 That poems could be a way to convey fact;

 By splitting sample researchers in two:

 And giving each a different abstract.

 The findings showed that whilst prose was preferred,

 Related meanings from both were inferred.   

 In keeping with the findings of this study, I think that offering both prose and poetry would be most suitable.

*Within the introduction, I enjoyed reading about the power and placement of a research article and what it represents to the reader. As the author suggests the abstract is often the decision-making part of the article: do you read further or purchase the article or do you pass it by, and, I know in myself that I have often been guilty of regarding the abstract as the last step prior to submission. To me, the power of this article has been to remind me to spend more time on this aspect of writing a research paper.*

 I am really glad to hear that this is the case, and since I conducted this study I have also found this to be the case with my other research papers. The abstract is the advert to the outside world in many respects, and so it deserves much more respect than as an afterthought, for the benefit of both the reader(s) and the author(s).

*Within the discussion and the conclusions, the concept, that poetry may serve as a useful abstract I still have some outstanding questions that I would like to have addressed. First of all, did the author consider producing two or three versions of the poem? The poem did seem to be less factually intense than the original abstract and I wonder if the authors feel that this may be reflected in the views of the scientists.*

 I agree entirely that different versions of the poems, ideally from different authors, would potentially illicit different responses. In the final paragraph I write that “Likewise, the responses of the participants would probably be different if they were shown different poetical interpretations (from the same or different authors) of the same abstract.” Which I think illustrates my agreement with you. When this study was designed I was unsure what the results would be, and was also unsure of what effect the choice of poem would have on the audience. However, analysing the results of this study, and reflecting on the process has led me to confirm that it would be beneficial to have a number of different poems offered as an alternative to the reader. I have now made this clearer in the conclusions.

*I am also aware that the beauty of a poem often lies in the critical analysis that underpins the choice of word and form used by the author. This is a skill that can be developed often throughout a school of HE education and I am not sure that many scientists will have received that training. However again this is my conjecture and not necessarily based on fact. The suggestion is that scientists are happier to read a prose abstract. I also wonder if this also reflects many scientists concern with an issue of whether they might have to learn to write an abstract as a poem.*

 Whilst I take your point, it is not the ‘beauty’ of the poem that is being called into question here, but rather its ability to summarise the key findings of the study, and to entice the reader into wanting them to find out more. The poem need not be beautiful in order to do that. In the penultimate paragraph of the ‘results and Discussion’ section I write that “Could it also be that the participants were on the whole less experienced in analysing poetry, and therefore felt less confident in elucidating on their opinions as to the nature of the poem?”, which aligns with the point that you make above regarding scientists and their happiness of reading a prose vs. a poem abstract. This is now further elucidated in the ‘Conclusions’, as is your excellent suggestion regarding the fear of scientists maybe having to write poetry!

*I agree with the author that the abstract chosen was well written and accessible. I think that this is the key. Prose or poetry is not the issue but accessibility is and writing that can be clearly understood has to be key to this. The author suggests that they would like to repeat the  study of a more 'general' audience. I can see the value of this approach and the importance of this study as it is important to explore how science can be made more accessible to a more general public. I wonder whether the author would consider using three versions of an abstract in future studies. The first an abstract written for a scientific community but not necessarily a more general audience, the second an abstract that is written in a clear accessible language and the last a poem. I also think that a lot more value would be placed in having a variety of abstracts presented in this way to get a more robust data set.*

 This is an excellent suggestion, and one which I have now incorporated into the ‘Conclusions’. Thank you. I have also changed the phrase ‘general audience’ to ‘non-expert’ audience, as I thought it to be more appropriate.

*I was also looking for a more thorough explanation for why the author only chose to present one poetry and prose abstract within this study.*

 This was simply as a result of limited resources. As specified in the text, this study was to see if there was a capacity and a merit in such investigations, which I think has been demonstrated. Based on the findings of this study, the planned future research with a more general audience will contain a much larger variety of both prose and poetic abstracts.

*For my own use, how I would use this article in the future is to consider bringing this into my own teaching practice. I think that a student that can read and summarise an article effectively in a poem may be demonstrating some key academic skills that include reading and digesting an academic piece of writing, being able to summarise effectively, being able to use effective writing to write for a less scientifically literate audience.*

 Thank you for your comments, and I would be very interested to see the results of such classroom activities, which I also use in my own teaching practices.

# Response

**Published:** 2016-09-19

**Authors:** Laura Bowater

**Affiliations:** University of East Anglia, UK

Thank you for your responses to the referee report. I agree that the poem is a welcome addition to the article and I feel that all my points have been addressed. Thank you for your response.

# Peer review: Referee response for version 2

**DOI:** 10.5256/f1000research.9197.r13973

**Published:** 2016-05-27

**Authors:** Katherine Rowan

**Affiliations:** 1 Professor and Director Graduate Program in Science Communication, George Mason University, Fairfax, VA, USA

I have read the new version of Illingsworth's paper and am happy to approve its indexation, with suggestions for the following two minor revisions.

 First, I believe the author intended to use the word, **elicit**, not illicit, in the sentence: "As well as not conveying some key elements of the study (as was the case with the ‘Model’ category) there is also the danger that the poem might **illicit **in the reader an implied meaning".

 Second, I would be happier if the writer also cited following:

Rowan, K. E. (1988).  A contemporary theory of explanatory writing. ***Written Communication***, ***5***, 23-56.

Rowan, K. E. (2003).  Informing and explaining skills:  Theory and research on informative communication.  In J. O. Greene & B. R. Burleson (Eds.), ***The handbook of communication and social interaction skills*** (pp.  403-438).  Mahwah, NJ:  Erlbaum.

 The Rowan (1988, 2003) papers argue, following James Kinneavy's (1971) *Theory of Discourse*, that scientific discourse and literature discourse have differing goals. Science is designed to provide empirical support for claims representing some aspect of reality. Literary discourse, which includes poetry, is designed to edify or entertain. My main concern with offering poetry in place of a scientific abstract is that poetry is not intended to provide a proof or evidence that something is the case about the natural or social world. That is the job of scholarly discourse. But it is possible Professor Illingsworth and I will disagree about this matter, so my sense is that I should raise this point one more time, but not let it stand in the way of this interesting paper being indexed.

I have read this submission. I believe that I have an appropriate level of expertise to confirm that it is of an acceptable scientific standard.

# Response

**Published:** 2016-08-18

**Authors:** Samuel Illingworth

**Affiliations:** Manchester Metropolitan University, UK

*I have read the new version of Illingworth’s paper and am happy to approve its indexation, with suggestions for the following two minor revisions.*

 Thank you for comments and review, which I now respond to on a point-by-point basis.

*First, I believe the author intended to use the word, elicit, not illicit, in the sentence: "As well as not conveying some key elements of the study (as was the case with the ‘Model’ category) there is also the danger that the poem might illicit in the reader an implied meaning".*

 Thank you for pointing this out. It has now been corrected.

*Second, I would be happier if the writer also cited following:*

*Rowan, K. E. (1988).  A contemporary theory of explanatory writing.  Written Communication, 5, 23-56.*

*Rowan, K. E. (2003).  Informing and explaining skills:  Theory and research on informative communication.  In J. O. Greene & B. R. Burleson (Eds.), The handbook of communication and social interaction skills (pp.  403-438).  Mahwah, NJ:  Erlbaum.*

*The Rowan (1988, 2003) papers argue, following James Kinneavy's (1971) Theory of Discourse, that scientific discourse and literature discourse have differing goals. Science is designed to provide empirical support for claims representing some aspect of reality. Literary discourse, which includes poetry, is designed to edify or entertain. My main concern with offering poetry in place of a scientific abstract is that poetry is not intended to provide a proof or evidence that something is the case about the natural or social world. That is the job of scholarly discourse. But it is possible Professor Illingworth and I will disagree about this matter, so my sense is that I should raise this point one more time, but not let it stand in the way of this interesting paper being indexed.*

 Thank you for these comments and references. I don’t think that we disagree entirely, but I think that poetry has the potential to maybe provide that information as a second-hand piece of evidence. I now comment on this further in the third paragraph of the ‘Survey Selection’, noting the following: “It is also important to consider that scientific discourse and literature have traditionally differing goals, with science designed to provide empirical support, and poetry (as a form of literary discourse) designed mainly to entertain (see e.g. Rowan, 1988, Rowan, 2003). With this in mind, this study sets out in part to see if poetry can in fact provide information about the natural world, by accessibly summarising a piece of scientific research.”

# Peer review: Referee response for version 1

**DOI:** 10.5256/f1000research.8377.r12267

**Published:** 2016-03-08

**Authors:** Katherine Rowan

**Affiliations:** 1 Professor and Director Graduate Program in Science Communication, George Mason University, Fairfax, VA, USA

I enjoyed reading this paper because it is clearly written and its core question, how should we make science accessible and compelling, matters.  I also appreciate knowing about the author’s blog. 

The author’s argument is that it is possible to transform a scientific abstract into accessible and compelling poetry and that the poetic version can be effective, accessible, and compelling.  I think he offers a demonstration that suggests this may be possible.  However, this argument is somewhat like saying you can transform a tractor into a bicycle and still retain many of the tractor’s key features.  You can, but the process of re-engineering the tractor as a bicycle changes not only its form but also its function.  Similarly, re-writing an abstract as poetry is not a translation.  It’s a transformation.  The author transformed the goal of the abstract when he rendered it as poetry. 

I think this paper **should **pass peer review, if it discusses the goals that animate science versus the goals that animate popularization.  Here are some resources that may be useful for this discussion.  Jane Martin (1970) distinguishes explanation to prove (science, scholarship) from explanation to teach (popular science, textbooks).  James Kinneavy (1971) makes a similar argument, saying scholarship and journalism are both forms of reference discourse, text designed to represent reality. Scholarship is primarily designed to present proof or evidence for its claims; journalism and popularizations aim to make research accessible and compelling.  In Kinneavy’s theory, poetry is viewed as literary discourse, text designed to edify and entertain.  I extend Kinneavy.  In a series of papers, I (Rowan, 1988, 1989, 1990, 2003) distinguish texts designed to create awareness (headlines, weather reports, sport scores) from texts that deepen understanding (explanatory science features, textbooks).   When Illingsworth casts a scientific abstract as poetry, he changes the text’s primary goal.  It no longer is focused on offering careful evidence for a claim, but on making the text’s claim accessible and compelling.  There should be a section in this paper noting this shift. 

One other limitation to the study is its design.  Currently, the design involves a sample of one:  one study.  Ideally, there should be 75 or so studies and poetic presentations of their abstracts. This ideal design would avoid confounding the effects of this one study with the effects of interest (does a poetic account make a scientific abstract more accessible and compelling). Realistically, Illingsworth has already done substantial work in presenting the evidence he did concerning one abstract and its poetic version’s effects.  Perhaps three to five abstract-poetry pairs would be a more realistic request for the study’s design.  Then, there would be a chance to observe whether the topic of a study has an impact on the chances of rendering its abstract in poetry.  Regardless, I think this paper is so well written and its topic so important that I hope a revised version, with the limitations or concerns noted here, is published.

I enjoyed learning about Illingsworth’s work and will share his paper with my students in science journalism.

I have read this submission. I believe that I have an appropriate level of expertise to confirm that it is of an acceptable scientific standard, however I have significant reservations, as outlined above.

## References

[1] MartinJ: Explaining, understanding, and teaching.1970;New York: McGraw-Hill:

[2] KinneavyJ: A theory of discourse.1971;New York: W. W. Norton:

[3] ROWANK: A Contemporary Theory of Explanatory Writing. *Written Communication*.1988;5(1) : 10.1177/0741088388005001002 23-56 10.1177/0741088388005001002

[4] ROWANK: Cognitive Correlates of Explanatory Writing Skill: An Analysis of Individual Differences. *Written Communication*.1990;7(3) : 10.1177/0741088390007003002 316-341 10.1177/0741088390007003002

[5] RowanK: Moving Beyond the What to the Why : Differences in Professional and Popular Science Writing. *Journal of Technical Writing and Communication*.1989;19(2) : 10.2190/2V4E-G0XU-2K4P-UQH0 10.2190/2V4E-G0XU-2K4P-UQH0

[6] RowanK.E: Informing and explaining skills: Theory and research on informative communication.2003;Mahwah, NJ: Erlbaum:403-438

# Response

**Published:** 2016-04-01

**Authors:** Samuel Illingworth

**Affiliations:** Manchester Metropolitan University, UK

*I enjoyed reading this paper because it is clearly written and its core question, how should we make science accessible and compelling, matters.  I also appreciate knowing about the author’s blog.*

Thank you very much for these kind comments. I am very glad that you enjoyed reading the paper, and that you found the subject matter to be important.

*The author’s argument is that it is possible to transform a scientific abstract into accessible and compelling poetry and that the poetic version can be effective, accessible, and compelling.  I think he offers a demonstration that suggests this may be possible.  However, this argument is somewhat like saying you can transform a tractor into a bicycle and still retain many of the tractor’s key features.  You can, but the process of re-engineering the tractor as a bicycle changes not only its form but also its function.  Similarly, re-writing an abstract as poetry is not a translation.  It’s a transformation.  The author transformed the goal of the abstract when he rendered it as poetry.*

This is an excellent point, and the issue of transformation is discussed in more detail in the response to the following paragraph.

*I think this paper **should **pass peer review, if it discusses the goals that animate science versus the goals that animate popularization.  Here are some resources that may be useful for this discussion.  Jane Martin (1970) distinguishes explanation to prove (science, scholarship) from explanation to teach (popular science, textbooks).  James Kinneavy (1971) makes a similar argument, saying scholarship and journalism are both forms of reference discourse, text designed to represent reality. Scholarship is primarily designed to present proof or evidence for its claims; journalism and popularizations aim to make research accessible and compelling.  In Kinneavy’s theory, poetry is viewed as literary discourse, text designed to edify and entertain.  I extend Kinneavy.  In a series of papers, I (Rowan, 1988, 1989, 1990, 2003) distinguish texts designed to create awareness (headlines, weather reports, sport scores) from texts that deepen understanding (explanatory science features, textbooks).   When Illingworth casts a scientific abstract as poetry, he changes the text’s primary goal.  It no longer is focused on offering careful evidence for a claim, but on making the text’s claim accessible and compelling.  There should be a section in this paper noting this shift.*

I think that this is an excellent point, thank you so much for bringing this to my attention, and also for providing some very useful and extremely interesting references. The issue of the goals which animate science vs. those which animate popularization need to be discussed, and are presented in the introduction as such:

“It is also important to consider the issue of transformation, and how this potentially affects the goal of the abstract. As discussed above, the primary objective of the abstract is to both provide information and also to convince the reader to finish the remainder of the paper. The nature of the poem means that it is more likely to be thought of as a popular piece of science writing rather than a professional piece of science writing, as is the case for the original abstract. As such, care must be taken to ensure that in the transformation from prose into poetry, the primary objective does not also fully transform into that of purely establishing the novelty of the topic (Rowan, 1989). In order for the poem to still be useful to scientists it must still provide a useful summary of the research. However, could the poem do so in a more accessible manner than that of the more traditional scientific abstract?”

However, it is also worth noting that the primary goal of the scientific abstract is also to some extent to entice the reader, and to encourage them to read the rest of the article. Therefore, whilst the primary objective of the scientific article might be offering careful evidence for the claim, the primary objective of the scientific abstract is to offer an accurate summary which also compels the reader to continue to the rest of the article. The primary objectives of the abstract are discussed in more detail in the revised Introduction to the manuscript, as discussed in the response to Referee 2. Whilst I agree that I have transformed the abstract by presenting it as a poem, I did so in such a way that I tried to still preserve the scientific sense of the article in question. This study was also focused on whether or not researchers would still find the poem to be accessible in terms of how useful it was in conveying scientific information to them, and as I outlined in the Introduction, the purpose of this study was to see if a poem was still “accessible and informative to other scientists.” As such I believe that there was a shift, but maybe not to the extent that you have outlined above. However, the fact that there is a shift at all is a very important point, and as such has now been addressed in the discussion, with the addition of the following text:

“As discussed in the Introduction, the very nature of this study involves transforming the abstract in some way, and whilst every effort has been made in this transformation to retain the information of the original abstract, it is clear from an analysis of the surveyed responses that this has not been entirely successful. This is most clearly evidenced with the omission of the word ‘model’ from the poem. Whilst it is likely that the results would have been different had the first line of the poem been replaced with “In Canada a model found,” this is not the transformation that the author decided upon. Has the text’s primary goal therefore also changed? As discussed in the Introduction, the primary goal of the scientific abstract is to offer an effective summary of the study, but also to compel the reader, in this instance to read the rest of the article. The results of the survey would suggest that rather than a transformation of primary goal, there has instead been a transformation of focus. As with the original abstract, the purpose of the poem was still to inform *and* entice scientists, by presenting an indicative summary of the underlying research, and also serving as a compelling case that the remainder of the article was worthy of the reader’s attention. The analysis of the qualitative data would seem to suggest that the poem has still served that purpose (seemingly neither improving nor diminishing the desire to find out more about the study), however the focus of the summary has undoubtedly shifted.”

The issue of transformation was also further addressed in the conclusions, with the following text:

“The issue of transformation that was raised in the discussions is notable, and is certainly worthy of further investigation in future studies. However, such studies would need to be designed so as to specifically address this issue. For example, participants could be asked for their interpretations of the primary objective of each version of the abstract (poetry or prose). Alternatively, participants could be shown both versions of the abstract and could be asked to comment on similarities and differences between the two, both literally and in terms of what they convey.”

*One other limitation to the study is its design.  Currently, the design involves a sample of one:  one study.  Ideally, there should be 75 or so studies and poetic presentations of their abstracts. This ideal design would avoid confounding the effects of this one study with the effects of interest (does a poetic account make a scientific abstract more accessible and compelling). Realistically, Illingworth has already done substantial work in presenting the evidence he did concerning one abstract and its poetic version’s effects.  Perhaps three to five abstract-poetry pairs would be a more realistic request for the study’s design.  Then, there would be a chance to observe whether the topic of a study has an impact on the chances of rendering its abstract in poetry.*

I agree that the sample size is small. However, throughout the paper I have ensured that I have not made any generalizations, and I have acknowledged that there are clearly limitations in what can be derived from the study. Whilst conducting 75 studies would certainly yield more conclusive results, it is beyond the scope and indeed the resources of this study, which was carried out without any funding. Part of the purpose of this study was to demonstrate that there is an interest and a capacity for such investigations, which can hopefully be used as leverage for further research, in which for example the issue of transformation can be more specifically addressed.

Regarding the use of three to five different poems and abstract comparisons, again I think that this would make for an interesting study, but that it would be beyond the scope and resources of this investigation. Furthermore, and pending future funding, I think it would potentially be more useful to repeat the study with three to five different groups of scientists using the same poem, as otherwise it may be difficult to analyse the data across the different poems. For example, all poems would probably have to be written in a fairly similar style and using a similar metric and structure, as otherwise there may be different reasons for the way in which the poem was ‘received’ by the participants. However, this is something that could also potentially be investigated in future studies, as could a comparison of different poems written by different authors, all of whom might have been given a different remit to perform their transformation under. There is a genuine wealth of possibilities that are available here for investigation, and I hope that this paper acts as a starting point for such studies, both by myself and ideally from other interested colleagues as well. Regarding the limitations of the study I have made further explicit reference to them with the following passage in the conclusions:

“It is acknowledged that the results and subsequent analysis that are presented here represent the responses from only one study that was carried out on a small subset of participants. As such, it is important to recognise the limitations of the findings, and to allow that a different set of results may be evident if a different group of participants were surveyed. Likewise, the responses of the participants would probably be different if they were shown different poetical interpretations (from the same or different authors) of the same abstract. Given these limitations, I hope that this study has demonstrated that there is a capacity and a merit in such investigations, and also that it has served as inspiration for future work. ”

*Regardless, I think this paper is so well written and its topic so important that I hope a revised version, with the limitations or concerns noted here, is published.*

Thank you. I hope that I have now addressed these issues in an acceptable manner.

*I enjoyed learning about Illingworth’s work and will share his paper with my students in science journalism.*

Thank you, that is greatly appreciated.

# Peer review: Referee response for version 1

**DOI:** 10.5256/f1000research.8377.r12381

**Published:** 2016-02-26

**Authors:** Magnus Johnson

**Affiliations:** 1 Centre for Environmental and Marine Sciences (CEMS), University of Hull, Scarborough Campus, Scarborough, UK

I would really like to see this paper pass peer review eventually as it is undoubtedly an interesting topic. However, in my opinion, it needs a lot of work to knock it into shape and they may even change the conclusions.

I think a bit more here on what an abstract should be (or is) and previous research on the clarity of abstracts would be useful. An abstract, at its most fundamental, can consist of 4 sentences:

1. What are you interested in
2. How did you study it
3. What did you find
4. Why is that interesting?

It should be completely clear, written in simple language and understandable to those outside the research field and hopefully to the general public.

You could take any abstract from Science or Nature and it would often not be understandable by a lay-person, or most scientists that are not active in the particular field that the paper is about.  You have chosen an example that is relatively good, I wonder if you had presented folk with something a little more dense whether you would have got the same result?

The dataset is much richer than suggested by your simple analyses. I would like to have seen some analysis of demographics v response - otherwise why present the data in your supplemental?  Do men and women respond in the same way?  What about the proximity of someone’s own topic/job/role to that of the abstract you provided. I like John Wedgewood Clarke’s poem on Marine Protected Areas ^1^, and find it a useful tool for contemplation but is that because it is an area close to my heart? 

Could the font on the boxplots be enlarged so that the figures are legible without having to download the paper/slide?

The non-parametric equivalent of central tendency is a median rather than a mean.  You are using the appropriate figure (boxplots) which give the median, 25 & 75% quartiles and 95% confidence interval.  You then give means and standard deviations in table 1 – these are not really appropriate for non-parametric data.

The probability distributions of data in different categories does impact on how you interpret your results. https://statistics.laerd.com/premium-sample/mwut/mann-whitney-test-in-spss-2.php

Lots of mixing of parametric and non-parametric descriptions of data – you need to avoid that.  I’m not sure there is any reason why you should assume normality of the data so non-parametric would be the logical way to go.  The length of responses v prose/poetry analyses are problematic in my opinion. Does their length reflect the length of the abstract/poem? The poem necessarily contains less detail. You may have got a different response had you asked – “why is this piece of work important” where the responded had to contemplate on rather than just reflect the contents. 

The discussion is very weak.  I would expect to see the results reflected in the literature.  You may be better splitting the results and discussion section into two parts. The fact that folk responding to the poem did not use the word “Model” is hardly surprising when the word is not in or directly implied in the poem. Had the first line been “In Canada a model found” the result would undoubtedly be different.

I have read this submission. I believe that I have an appropriate level of expertise to confirm that it is of an acceptable scientific standard, however I have significant reservations, as outlined above.

## References

[1] Wedgewood ClarkeJ: The Sea Addresses an MPA. *Adv Mar Biol*.2014;69: 10.1016/B978-0-12-800214-8.09981-3 xxv 10.1016/B978-0-12-800214-8.09981-3 Reference source

# Response

**Published:** 2016-04-01

**Authors:** Samuel Illingworth

**Affiliations:** Manchester Metropolitan University, UK

*I would really like to see this paper pass peer review eventually as it is undoubtedly an interesting topic. However, in my opinion, it needs a lot of work to knock it into shape and they may even change the conclusions.*

*Thank you for your comments, which have proven to be extremely useful. Please see below for a discussion of the work that has been done in order to respond to your comments.*

*I think a bit more here on what an abstract should be (or is) and previous research on the clarity of abstracts would be useful. An abstract, at its most fundamental, can consist of 4 sentences:*

1. *What are you interested in*
2. *How did you study it*
3. *What did you find*
4. *Why is that interesting?*

*It should be completely clear, written in simple language and understandable to those outside the research field and hopefully to the general public.*

Thank you for raising this issue. I agree that more could have been written about the purpose of the scientific abstract, with the following text now included in the introduction:

“Andrade (2011, pp. 172) notes that “for the vast majority of readers, the paper does not exist beyond its abstract,” with the majority of researchers using the abstract to determine if the scientific study is relevant to them and worthy of a further investment of their time in reading it in its entirety. As noted by Fletcher (1988),  the creation of an abstract is often also an extremely important process for clarifying the narrative of the scientific study in the mind of the author(s) themselves. Hartley (2003) also found that structured abstracts (i.e. those split into subheadings of: Background, Aims, Methods, Results, and Conclusions, or their equivalents) were found to be more informative and also provide greater clarity than their unstructured counterparts.  Whilst the exact format and structure of the abstract will be determined by the journal in which the article is to be published, the purpose of writing an abstract should be to extract and summarize (Alexandrov and Hennerici, 2006), with the primary objective to not only provide information, but also to convince the reader to finish the remainder of the paper, which in some instances will involve paying for the privilege (Koopman, 1997).

Orasan (2001) also observed that many authors of scientific papers do not consider the abstract to be particularly important, arguing that in many cases it is written as a necessity just before the paper is submitted. Is it therefore the case, that rather than being an effective and economical method of communicating the research, that the abstract instead represents a rushed précis of the research findings, with an even higher lexical density than that of the main body of the text? If so, then are they useful to anyone who might consider themselves, or indeed be considered an outsider? And if non-experts are unable to fully grasp the summary of the explanation, then what hope do they have of being able to fully understand the research and its potential relevance to them?  Cross and Oppenheim (2006) also notes that there is probably little formal training in abstract writing, which is why in some instances there may be a lack of clarity in the abstracts that are produced in scientific journal articles.”

I have specifically tried to not advise on the layout of an abstract, because as mentioned in the above text this is something that is specific to the journal. However, I hope that the above addition, as well as the additional information in regards to the primary objective of the scientific abstract seek to better highlight previous work that has been done in relation to the role of the abstract.

*You could take any abstract from Science or Nature and it would often not be understandable by a lay-person, or most scientists that are not active in the particular field that the paper is about.  You have chosen an example that is relatively good, I wonder if you had presented folk with something a little more dense whether you would have got the same result?*

I agree that the result may have been different if a different article had been presented to the participants. However, this is beyond the scope of this study, and in this instance the abstract was specifically chosen so as not to negatively bias the study against the original prose. This is explained further in the revised manuscript, with the inclusion of the following text in the Survey Selection section:

“It was also important that the abstract that was chosen was itself well written, and that it met the primary objectives of an abstract that was described in the Introduction, i.e. that it presented a clear and accurate summary of the paper, and left the reader wanted to find out more. Whilst this latter point is relatively subjective, it was the author’s opinion that this abstract did indeed meet these primary objectives, thus the reason for its selection. If a less clear or less obviously enticing abstract had been chosen then there was a risk that the study might be being perceived as negatively biased towards the prose version of the abstract. It is acknowledged that in choosing such an effective abstract the study might instead by positively biased towards the original prose, but given the nature of the investigation it was felt that this was more appropriate.”

*The dataset is much richer than suggested by your simple analyses. I would like to have seen some analysis of demographics v response - otherwise why present the data in your supplemental?  Do men and women respond in the same way?  What about the proximity of someone’s own topic/job/role to that of the abstract you provided. I like John Wedgewood Clarke’s poem on Marine Protected Areas* *^1^* *, and find it a useful tool for contemplation but is that because it is an area close to my heart?*

Thank you for this comment. The data was presented in the supplementary analysis because I wanted it to be made available to encourage other researchers to use it, as I agree that there is some demographic data there that may be useful for a future study. However, given the limitations of this particular study (which are now more explicitly discussed in the Conclusions), I don’t believe that it would add anything further to the analysis of the data, in terms of the expectations that were set out in the Introduction. As explained in the response to Referee 1, this study was carried out without any funding, and part of its purpose was to demonstrate that there is an interest and a capacity for such investigations, thereby hopefully generating both future funding and also inspiration for further studies.

*Could the font on the boxplots be enlarged so that the figures are legible without having to download the paper/slide?*

Thank you for pointing this out, I have now enlarged the font considerably.

*The non-parametric equivalent of central tendency is a median rather than a mean.  You are using the appropriate figure (boxplots) which give the median, 25 & 75% quartiles and 95% confidence interval.  You then give means and standard deviations in table 1 – these are not really appropriate for non-parametric data.*

*The probability distributions of data in different categories does impact on how you interpret your results.* *https://statistics.laerd.com/premium-sample/mwut/mann-whitney-test-in-spss-2.php*

*Lots of mixing of parametric and non-parametric descriptions of data – you need to avoid that.  I’m not sure there is any reason why you should assume normality of the data so non-parametric would be the logical way to go.*

Thank you for pointing this out, and I apologize for any confusion that was brought about by this mixing. All of the statistics in Table 1 have been replaced with median values, as recommended. In addition to that the text now reflects the non-parametric analysis that was carried out. I have left in the information relating to the Shapiro-Wilk test, as I believe that is important to statistically demonstrate that a non-parametric approach was appropriate, so that all readers could appreciate this.

*The length of responses v prose/poetry analyses are problematic in my opinion. Does their length reflect the length of the abstract/poem? The poem necessarily contains less detail. You may have got a different response had you asked – “why is this piece of work important” where the responded had to contemplate on rather than just reflect the contents.*

Thank you for raising this point, as it is important that it is addressed in the text. I believe that the brevity in the responses to the poem (in comparison to the prose) may in part be due to the participant’s relative lack of expertise in analyzing poetry. Just because a poem is shorter does not necessarily mean that it contains less detail, although this detail might not necessarily be explicit. I have now raised this issue in the text with the following commentary:

“Could it be that the longer length of the prose abstract, combined with the expectancy of the readers in terms of what a scientific abstract should look like, meant that the prose format was able to convey more information and was therefore more accessible, encouraging more verbose and detailed summaries from the participants? Could it also be that the participants were on the whole less experienced in analysing poetry, and therefore felt less confident in elucidating on their opinions as to the nature of the poem? Whilst the poem is shorter than the prose and contains less statistical information, does that necessarily mean that it contains less detail? Could it be that instead of explicitly communicating detail (as is the case with the prose abstract), the poem instead had the affect of implying detail via an emotive response or reflection by the reader? That only one of the participants commented on the emotive nature of the poem suggests that in this instance it may not be the case, and that for the participants of this study there probably was a perceived lack of detail in the poem compared to the prose. However, as this was not commented on (nor asked by the survey) explicitly this cannot be confirmed.”

*The discussion is very weak.  I would expect to see the results reflected in the literature.*

I respectfully disagree with this comment. I think that a detailed discussion has been given which both analyses the quantitative and qualitative data, and addresses the aims that were set out in the Introduction. I was unable to find any literature that would have been appropriate to compare to in the discussion, as I believe that this study is the first of its kind.

*You may be better splitting the results and discussion section into two parts.*

I think that in this instance it is better to group the results and discussion into one section, as the current layout of the paper allows a stronger narrative to be told, with the quantitative analysis leading onto a presentation of the qualitative data and then an analysis of this.

*The fact that folk responding to the poem did not use the word “Model” is hardly surprising when the word is not in or directly implied in the poem. Had the first line been “In Canada a model found” the result would undoubtedly be different.*

Thank you for raising this issue, as it was not properly addressed in the original manuscript. The following text has now been added to the end of the discussions section, which also includes a contextualization of the issue of transformation that is now addressed following a recommendation by Referee 1:

“As discussed in the Introduction, the very nature of this study involves transforming the abstract in some way, and whilst every effort has been made in this transformation to retain the information of the original abstract, it is clear from an analysis of the surveyed responses that this has not been entirely successful. This is most clearly evidenced with the omission of the word ‘model’ from the poem. Whilst it is likely that the results would have been different had the first line of the poem been replaced with “In Canada a model found,” this is not the transformation that the author decided upon. Has the text’s primary goal therefore also changed? As discussed in the Introduction, the primary goal of the scientific abstract is to offer an effective summary of the study, but also to compel the reader, in this instance to read the rest of the article. The results of the survey would suggest that rather than a transformation of primary goal, there has instead been a transformation of focus. As with the original abstract, the purpose of the poem was still to inform *and* entice scientists, by presenting an indicative summary of the underlying research, and also serving as a compelling case that the remainder of the article was worthy of the reader’s attention. The analysis of the qualitative data would seem to suggest that the poem has still served that purpose (seemingly neither improving nor diminishing the desire to find out more about the study), however the focus of the summary has undoubtedly shifted.”
